# MiMIC analysis reveals an isoform specific role for *Drosophila* Musashi in follicle stem cell maintenance and escort cell function

**DOI:** 10.1038/s41420-022-01245-5

**Published:** 2022-11-12

**Authors:** Nicole A. Siddall, Franca Casagranda, Timothy M. Johanson, Nicole Dominado, James Heaney, Jessie M. Sutherland, Eileen A. McLaughlin, Gary R. Hime

**Affiliations:** 1 Department of Anatomy and Physiology, The University of Melbourne, Parkville, VIC 3010 Australia; 2 Schools of Biomedical Science & Pharmacy, College of Health, Medicine and Wellbeing, University of Newcastle, Callaghan, NSW 2308 Australia; 3 Hunter Medical Research Institute, New Lambton Heights, NSW 2305 Australia; 4 Faculty of Science Medicine and Health, University of Wollongong, Gwynneville, NSW 2500 Australia; 5 School of Science, Western Sydney University, Penrith, NSW 2751 Australia; 6 Centre for Stem Cell Systems, The University of Melbourne, Parkville, VIC 3010 Australia; 7 Present Address: Walter and Eliza Institute of Medical Research, Parkville, VIC 3052 Australia

**Keywords:** Stem-cell niche, Differentiation

## Abstract

The *Drosophila* ovary is regenerated from germline and somatic stem cell populations that have provided fundamental conceptual understanding on how adult stem cells are regulated within their niches. Recent ovarian transcriptomic studies have failed to identify mRNAs that are specific to follicle stem cells (FSCs), suggesting that their fate may be regulated post-transcriptionally. We have identified that the RNA-binding protein, Musashi (Msi) is required for maintaining the stem cell state of FSCs. Loss of *msi* function results in stem cell loss, due to a change in differentiation state, indicated by upregulation of Lamin C in the stem cell population. In *msi* mutant ovaries, Lamin C upregulation was also observed in posterior escort cells that interact with newly formed germ cell cysts. Mutant somatic cells within this region were dysfunctional, as evidenced by the presence of germline cyst collisions, fused egg chambers and an increase in germ cell cyst apoptosis. The *msi* locus produces two classes of mRNAs (long and short). We show that FSC maintenance and escort cell function specifically requires the long transcripts, thus providing the first evidence of isoform-specific regulation in a population of *Drosophila* epithelial cells. We further demonstrate that although male germline stem cells have previously been shown to require Msi function to prevent differentiation this is not the case for female germline stem cells, indicating that these similar stem cell types have different requirements for Msi, in addition to the differential use of Msi isoforms between soma and germline. In summary, we show that different isoforms of the Msi RNA-binding protein are expressed in specific cell populations of the ovarian stem cell niche where Msi regulates stem cell differentiation, niche cell function and subsequent germ cell survival and differentiation.

## Introduction

**Fig. 1:**
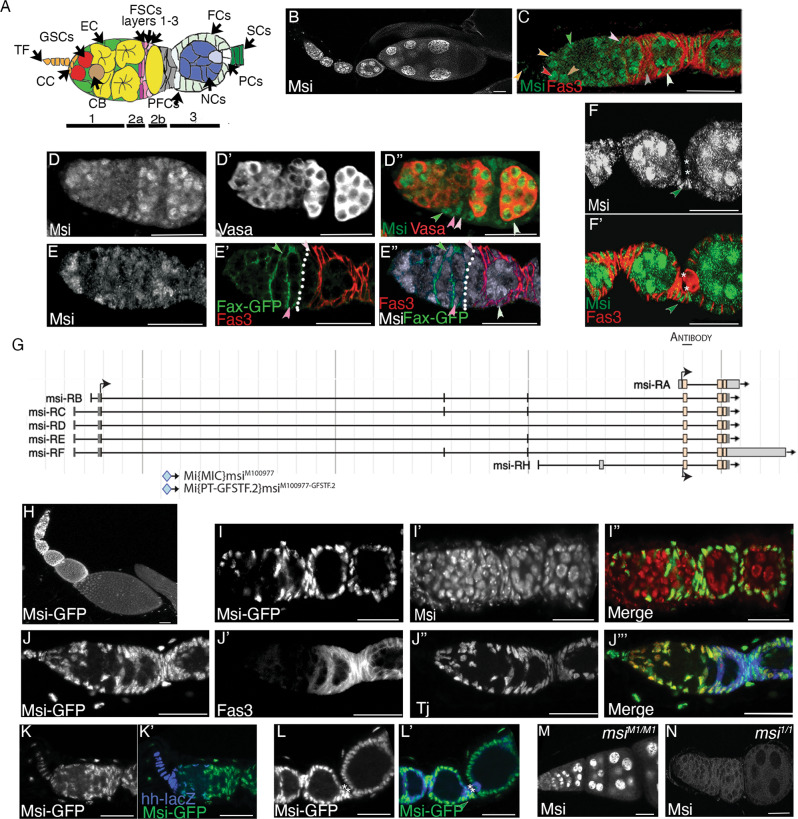
Differential expression of Msi isoforms in the adult ovary. **A** Cartoon depiction of the beginning stages of *Drosophila* ovary development showing the cell types, including terminal filament cells (TF, orange) and cap cells (CC, orange), escort cells (EC, green), germline stem cells (GSC, red), a cystoblast (CB, brown), germ cell cysts (yellow), follicle stem cells layers 1–3 (FSCs, layer 1 light pink, layer 2 medium pink, layer 3 mauve), pre-follicle cells (PFC, grey), polar cells (PC, white), nurse cells (NC, dark blue), differentiated follicle cells (FCs, light green) and stalk cells (SCs, dark green). **B**–**F**’ Confocal micrographs of Msi antibody expression in wild-type ovaries. **B** Low magnification micrograph of ovary showing Msi expression in the germline and somatic cells of the ovary. **C** Higher magnification micrograph of ovariole labelled with Msi (green) and Fas3 (red). Msi expression was observed in GSCs (red arrowhead), CBs (brown arrowhead), ECs (green arrowhead) and in somatic TFs and CCs (orange arrowheads). In this ovariole, a Msi-expressing layer 1 FSC is labelled (light pink arrowhead). Msi expression was observed in PFCs (grey arrowhead) and differentiated FCs (light green arrowhead). A reduction of visible Msi expression in 4-8 cell germline cysts was consistently observed. **D**-**D**” High magnification image of ovariole labelled with Vasa (red in **D**”) and Msi (green in **D**”). Msi-positive FSCs in layer 1 (light pink arrowhead) and layer 2 (medium pink arrowhead) are labelled. The green arrowhead labels a Msi-positive posterior EC, while the light green arrowhead points to a Msi-expressing differentiated FC. **E**-**E**” A confocal micrograph of an ovariole labelled with Msi (grey in **E**”), Fax-GFP (marking the membranes of ECs; green in **E**”) and Fas3 (red in **E**”) showing a Msi-expressing layer 1 FSC (light pink arrowhead) and layer 2 FSC (dark pink arrowhead) at the 2a/b boundary (white dotted line). **F**-**F**” Single-plane confocal image showing Msi expression (green in **F**’) in later stage egg chambers also labelled with Fas3 (red). Msi expression was observed in nurse cells, mature follicle cells and stalk cells (dark green arrowhead). * Denotes the polar cells, where Msi expression was barely detectable by immunofluorescence. **G** Image modified from Flybase (J-Browse) depicting 7 *msi* transcripts. Coding start sites are labelled (black arrow), but for representation only one arrow shows that isoforms (**B**–**F**) have the same coding start site. The genomic region of the peptide sequence used to generate the Msi antibody is labelled and the insertion site of Mi{MIC}msi^M100977^, referred to as the *msi*^*M1*^ allele in our paper, is shown. The Msi-GFP protein trap, also shown in this schematic, had been generated from the Mi{MIC}msi^M100977^ insertion. **H**–**N** Confocal micrographs mapping Msi-GFP expression in early oogenesis. **H** Low magnification projected image of Msi-GFP expression in the ovary shows GFP in the somatic cells. **I**-**I**” Confocal micrograph of ovariole labelled with the Msi antibody (red in **I**”) and Msi-GFP (green in **I**”) reveals that Msi-GFP is expressed in somatic cells but not the germline of the ovary. **J**-**J**”’ Projection of 3 single-plane confocal images from a z-stack shows Msi-GFP expression (green in **J**”’) completely overlaps with TJ-positive somatic cells (red in **J**”’) in the germarium and early-stage egg chambers. **K**-**K**” Msi-GFP expression (green in **K**’) overlaps with *hh*-lacZ expression (blue in **K**’) in TFs and CCs of ovarioles. **L**-**L**’ Msi-GFP (green in **L**’) is expressed in SCs (dark green arrowhead) and PCs (asterisks). **M** Single-plane confocal image of ovariole dissected from a *msi*^*M1/M1*^ homozygous adult shows Msi antibody expression in germ cells, but not somatic cells. **N** No Msi antibody expression was detected in ovarioles dissected from a *msi*^*1/1*^ null homozygous adult. Scale bars, 20 µm.

Adult stem cells have the ability to self-renew and give rise to differentiated cells in order to maintain tissue homoeostasis in multicellular organisms [1]. The adult *Drosophila* ovary is an excellent model for the study of stem cell behaviour and organ morphogenesis. Each ovary is comprised of 16–18 sequential chains of egg chambers (ovarioles), the most mature found furthest from the anterior germarium [2]. The germarium is divided into 3 regions (Fig. 1A). Region 1 germline stem cells (GSCs) reside within a niche of somatic cap cells (CC), terminal filament cells (TF) and escort cells (ECs). GSCs divide asymmetrically to produce daughter cystoblasts (CBs). CBs divide four times synchronously with incomplete cytokinesis to form mitotic cysts of 2, 4, 8 and 16 cells [2]. ECs, also known as inner germarial sheath (IGS) cells, engulf cystoblasts and germline cysts in region 1 and 2a [3]. Follicle cells (FCs), derived from follicle stem cells (FSCs), surround cysts in region 2b, and stage 1 egg chambers form in region 3 [2]. Tight associations of ECs with the germline are necessary to support germline development [3–6]. ECs differ in shape, size and ability to associate with germ cells depending on their germarial position, suggesting functional diversity [3]. Recent single-cell analyses have uncovered distinct EC subpopulations which interact with different developmental stages of GSC progeny and have distinct functions in the regulation of germline development [7–10].

Somatic FSCs reside in region 2a/2b of the germarium [11], the exact number of which remains controversial. Early studies proposed that 2–3 FSCs divide to give rise to a daughter stem cell and follicle precursor cell (FPCs). FPCs then differentiate into posterior follicle cell (FC) types, including polar, stalk and main body epithelial cells [11]. A recent study proposed that a population of approximately 14 FSCs exist in three layers within region 2a/2b [12]. This latter study, which utilised a novel lineage tracing system, suggested that FSCs in the most posterior layer (layer 1) give rise to the FC lineage, while cells within layer 3 could differentiate into more anterior ECs. The complexities of identifying a distinct population of FSCs cannot be understated, highlighted by the inability of scRNAseq to define a clear FSC population [9]. It has been suggested that this may be because the FSC gene expression profile is likely to be similar to pre-follicle cells, or that mitotic activity between FSCs and ECs is not significantly different [7, 9]. The lack of clarity in the identification and function of FSCs and surrounding somatic cells highlights the need for the identification of individual genes that affect differentiation of these cell types.

Numerous studies report the importance of RNA-binding proteins (RBPs) as essential regulators of stem cells in diverse organisms. In *Drosophila*, RBPs regulate the GSC lineage in both males and females [13–17]. We previously demonstrated an intrinsic requirement for the RBP Musashi (Msi) in GSC maintenance in the *Drosophila* testis [14]. In the *Drosophila* midgut, overexpression of Msi promotes stem cell proliferation after radiation-induced damage [18]. Its vertebrate orthologues, Msi-1 and Msi-2, function in the regulation, proliferation and maintenance of stem cells in multiple tissues [19–26] and play key roles in vertebrate spermatogenesis [27, 28] and folliculogenesis [29]. The recent discovery of isoform specific functions for Msi-1 or Msi-2 in processes such as tumour progression [30, 31] has added additional complexities to understanding the role of Msi proteins in developmental processes.

According to Flybase (FB2022_02), *Drosophila* Msi has 7 transcripts encoding 5 unique polypeptides [31]. To date there are no reports of *Drosophila* Msi isoform specificity in developmental processes. By using flies carrying a Minos-Mediated Integration Cassette (MiMIC) insertion in only a subset of Msi isoforms, and a GFP protein-trap (recombineered from the MiMIC transposon), we have uncovered Msi isoform expression differences in the ovarian soma and germline. We demonstrate an isoform specific requirement for Msi in ovarian somatic cells, with loss of Msi causing a loss of FSCs and precocious expression of a differentiation marker, Lamin C, in pECs and FSCs. We demonstrate that loss of Msi from ECs results in a failure of these cells to properly support germline cyst progression and an increase in region 2b germline cyst apoptosis. In contrast, we found no requirement for Msi isoforms in female GSC regulation, despite the shorter isoform/s being required for maintaining spermatogonial GSC fate [14], revealing sex-specific functions for Msi. Importantly, our study is the first to identify a functional requirement for alternative Msi isoforms in different *Drosophila* stem cell populations.

## Results

### Antibody and protein trap analysis reveal differential expression of Msi isoforms in the adult ovary

Analysis of Msi protein distribution in the ovary using a polyclonal antibody (Fig. 1N, Supplementary Fig. 1A) [14, 32] revealed expression in germline and somatic cells (Fig. 1B–F’). In the germarium, Msi was observed in GSCs and differentiated germ cells (Fig. 1B–D). Similar to our observations in male germ cells [14], a reduction of Msi protein expression levels was consistently observed in 2–4 cell cysts (Fig. 1C–E”). Msi expression persisted in nurse cells in later stage egg chambers but was absent from the oocyte (Fig. 1B). In somatic cells, Msi expression was observed in TFs, CCs, ECs and FSCs (Fig. 1C–E”), differentiated FCs and stalk cells (Fig. 1C–F’), but was barely detectable in polar cells (Fig. 1F-F’). FSCs were identified as somatic cells located at the region 2a/2b junction, with the most posterior layer 1 cells located at the Fas 3 boundary (Fig. 1C–E) [33].

Protein traps serve as an additional means to analyse expression patterns of proteins. A Msi-GFSTF (Msi-GFP) protein trap line, generated by recombination mediated cassette exchange (RMCE) from a Mi{MiC}msi^M100977^ insertion in a coding intron of 5 of the 7 *msi* transcripts was available from Bloomington Stock Centre. Msi-GFP recapitulates expression of longer Msi isoforms, but not shorter isoforms Msi-PA and Msi-PH. Conversely, the polyclonal antibody generated against amino acids 1–210 of Msi-PA detects expression of all isoforms [32] (Fig. 1G, Supplementary Fig. 2). Analysis of Msi-GFP yielded the discovery of GFP expression in only somatic cells of the ovary (Fig. 1H–L’). Msi-GFP expression overlapped with antibody labelling in somatic cells (Fig. I-I”) but was absent in the germline. All somatic cells labelled with anti-Traffic Jam (TJ) [34], which marks CCs, ECs, FSCs and progeny, co-expressed GFP (Fig. 1J-J”’). GFP expression was detected in *hh-lacZ* labelled TF cells (Fig. 1K-K’) and in stalk and polar cells (Fig. 1L-L’) despite Msi antibody labelling being undetectable in polar cells. This is likely due to the augmented ability to detect GFP by indirect immunofluorescence. These results reveal a divergence of expression of short Msi isoform/s (Msi-PA and/or Msi-PH) and the longer Msi isoforms in the ovary.

### Analysis of Msi expression in *msi* MiMIC mutants reveals a divergence of Msi isoform usage in the germline and somatic cells of the *Drosophila* ovary and testis

To understand the nature of the Mi{MIC}msi^M100977^ insertion (which from hereon will be referred to as *msi*^*M1*^) and determine its effect on ovarian Msi expression, we tested for the presence of Msi protein expression in *msi*^*M1*^ homozygotes. MiMIC insertions, when inserted in coding introns in the same orientation as the transcript, function as gene traps that can disrupt gene function [35]. *msi*^*M1*^ homozygotes are viable as adults but the presence of the MiMIC insertion is expected to disrupt expression and function of the longer Msi isoforms (Fig. 1G). In *msi*^*M1/M1*^ mutants, Msi antibody expression persisted in germ cells, but no expression was detected in somatic cells (Fig. 1M), thus demonstrating loss of Msi in the somatic cells of *msi*^*M1/M1*^ mutants, but not the germline. These data suggest a divergence of Msi isoform usage in the germline and somatic cells of the ovary. Msi expression divergence was also observed in the testis. Msi antibody labelling in the testis is detected in somatic hub cells, cyst progenitor cells, in GSCs and throughout the germline [14]. In *msi*^*M1/M1*^ testes, Msi antibody labelling persisted in the germline and early cyst cells but was absent from hub cells and mature cyst cells, suggesting that testis germ cells express Msi-RA/RH but not the longer isoforms (Supplementary Fig. 3A). This correlated with Msi-GFP expression, which was observed in hub, cyst progenitor and cyst cells, but was absent from the germline (Supplementary Fig. 3B).

### Loss of Msi from somatic cells results in an increase in the number of germarial cysts, germline cyst collisions and fused egg chambers

**Fig. 2:**
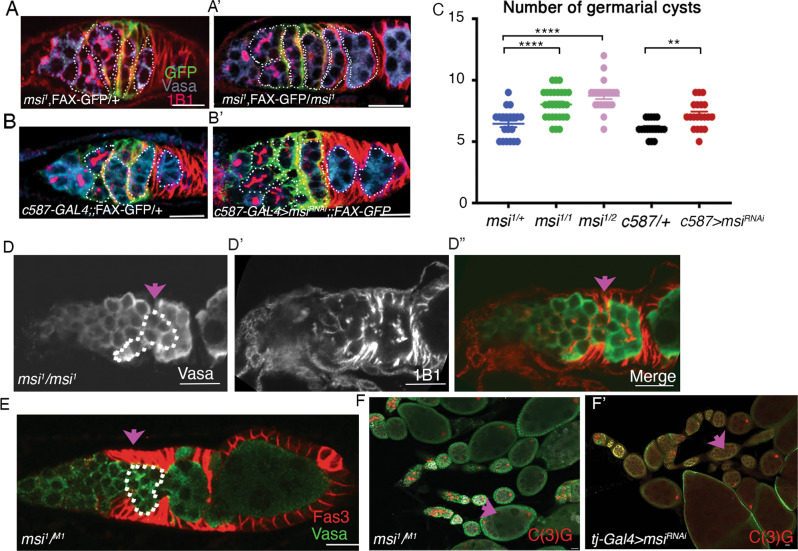
Loss of Msi from somatic cells results in an increase in the number of germarial cysts, germline cyst collisions in region 2a/2b and fused egg chambers. **A**, **B**’ Confocal micrographs of control (**A** and **B**), *msi* null (**A**’) and *C587-Gal4* > *UAS-msiRNAi* (**B**’) germaria. Germline cysts (dotted outlines) were counted in germaria labelled with Vasa (germ cells), 1B1 (fusomes showing connections between germ cells), and FAX-GFP, a marker of escort cell cytoplasm. **C** Scatterplot of the average number of 4–16 cell germline cysts in regions 1 to 3 of germaria (±SEM) dissected from heterozygote *msi*^*1/+*^ (blue; 6.45 ± .27; *N* = 20), *msi*^*1/1*^ (green; 8.04 ± .23; *N* = 28), transheterozygote *msi*^*1/2*^ flies (pink; 8.73^*±*^.27; *N* = 22), C587GAL4 > + (black; 6.11 ± .26) and C587GAL4 > msi^RNAi^ (7.2 ± .25). Welch’s two-tailed *t* tests reveal a significant increase in the average number of cysts in both *msi* null and *msi*^*1/2*^ mutants compared to control *msi*^*1/+*^ germaria (*p* < 0.0001 in both cases) and in C587-GAL4 > + control germaria (*N* = 18) compared to germaria dissected from C587-GAL4 > msi^RNAi^ flies (*N* = 20; *p* = 0.0012). Ovarioles were dissected from a minimum of 8 adult females from each genotype and ovarioles were imaged randomly over two separate sessions. **D**, **E** High magnification single-plane confocal micrographs show colliding cysts (pink arrow, dotted outline) in ovaries dissected from *msi*^*1/1*^ and *msi*^*1/M1*^ mutants. **F**–**F**’ Low magnification confocal micrographs of ovaries dissected from *msi*^*1/M1*^ and *tj*-GAL4 > msi^RNAi^ flies. Pink arrow points to fused egg chambers with two oocytes labelled with C(3)G. Scale bars, 20 µm.

We sought to investigate whether Msi is functionally required for early ovary morphogenesis. Homozygous *msi* null mutants (*msi*^*1/1*^) are viable into early adulthood, albeit being less fit than heterozygous and wild-type counterparts. Their ability to survive a few days, however, provides an opportunity to undertake morphological analysis by immunofluorescence in null mutants. The *msi*^*1*^ mutation was originally generated by imprecise excision of a P{LacZ} enhancer trap element near the 5’ region of *msi-RA* [36]. Msi antibody expression was not detected in *msi*^*1/1*^ null mutant ovaries (Fig. 1N) confirming loss of all isoforms. Germaria dissected from *msi*^*1/1*^ 2–3 day old adults were swollen in appearance and contained excess Vasa-labelled germ cells (Fig. 2A-A’). Using the fusome marker 1B1 and Vasa to identify germline cysts, we counted significantly more germarial cysts in *msi*^*1/1*^ ovarioles compared to control *msi*^*1/+*^ ovaries (Fig. 2C). A similar result was observed in ovaries from flies with a heteroallelic combination of the *msi*^*2*^ hypomorphic allele [36] (Supplementary Fig. 1A) and the *msi*^*1*^ null allele (Fig. 2C).

We sought to determine whether swollen *msi* mutant germarial cysts resulted from somatic or germ cell abrogation of Msi function. To this end, *C587-GAL4*, which drives expression in EC and early FCs [37], coupled with an UAS-*msi*^*RNAi*^ transgene (Supplementary Fig. 1B, C) was used to knockdown Msi function in somatic cells. Morphological analysis on ovaries dissected from 2–3 day old *C587-GAL4* > *msi*^*RNAi*^ and *C587-GAL4/* + adults revealed a significant increase in the number germarial cysts in *C587-GAL4* > *msi*^*RNAi*^ compared to control ovaries (Fig. 2B–C) suggesting a functional requirement for Msi in somatic cells to regulate early ovarian morphogenesis.

Analysis of *msi*^*1/1*^ mutants also revealed other morphological defects. In about 32% of *msi*^*1/1*^ ovaries (*N* = 28), germline cysts in region 2a-2b appeared to exhibit a cyst collision phenotype (Fig. 2D). Cyst collision is a process that occurs when forward moving germline cysts, or backward sliding cysts collide into the adjacent cyst [38]. This can occur from modification of the equilibrium between germline and somatic forces in germline cyst progression, for example, by decreasing germline contractility or adhesion, or blocking somatic cell movement. Both scenarios lead to the development of compound egg chambers [38]. Since Msi function is abrogated in both germline and soma of *msi*^*1/1*^ mutants, we assayed *msi*^*1/M1*^ transheterozygotes for evidence of cyst collision. Msi function in *msi*^*1/M1*^ flies is only perturbed in somatic cells and the heteroallelic combination circumvents any possibility of effects from second site mutations. *msi*^*1/M1*^ germaria labelled with Vasa in combination with Fas 3 revealed cyst collisions in 30% (*N* = 60) of *msi*^*1/M1*^ germaria (Fig. 2E). Since colliding cysts can result in formation of compound egg chambers [38], we labelled *msi*^*1/M1*^ ovaries with the synaptonemal complex marker C(3)G [39], which revealed the presence of compound egg chambers containing 2 oocytes in 10% of egg chambers (*N* = 40) (Fig. 2F). We confirmed that compound egg chambers were due to somatic loss of Msi function by combining UAS-*msi*^*RNAi*^ with the somatic cell driver *tj-GAL4*. 10% of *tj-GAL4* > UAS-*msi*^*RNAi*^ egg chambers (*N* = 42) were compound chambers with 2 oocytes (Fig. 2F’). Although Msi is expressed in all somatic cells in early oogenesis, and in the germline, our observations of excess germ cells and germline cysts, along with cyst collisions, was confined to region 2a/b in *msi* mutants. We confirmed the number of GSCs and cystoblasts expressing phospho-Mad (the expression of which is restricted to these cell types [37]) was not significantly different between control and *msi*^*1/1*^ ovaries (Supplementary Fig 4A-A’). Excess cysts were observed in regions 2a/b and 3, posterior to the Bag-of-Marbles (Bam) antibody expression domain, which labels 2–4 cell germline cysts [40] (Supplementary Fig. 4B, C). Together, our results suggest a functional requirement for Msi in region 2–3 somatic cells to control germline cyst morphogenesis.

### Msi is required for ovary FSC maintenance, but not GSC maintenance

**Fig. 3:**
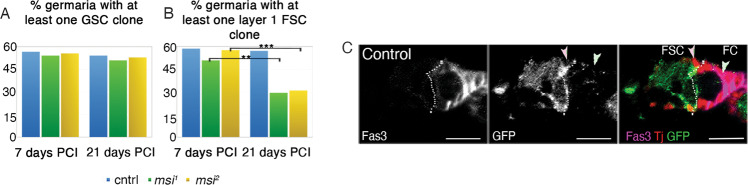
Msi is required for FSC maintenance, but not GSC maintenance. **A** The percentage of germaria containing at least on negatively marked control (blue), *msi*^*1*^ (green) or *msi*^*2*^ (yellow) GSC clone generated by Flp-FRT mediated recombination at 7 days PCI (56.7%, *N* = 97; 54.16%, *N* = 96 and 55.67%, *N* = 97 respectively) and 21 days PCI (54.16%, *N* = 96; 50.98%, *N* = 102 and 53%, *N* = 100 respectively). No significant differences were observed between genotypes as measured by Fisher’s exact test. **B** The percentage of germaria containing at least on negatively marked control (blue), *msi*^*1*^ (green) or *msi*^*2*^ (yellow) layer 1 FSC clone generated by Flp-FRT mediated recombination 7 and 21 days PCI. A significant reduction in the frequency of *msi*^*1*^ (29.41%, *N* = 102; Fisher’s exact test *p* = 0.0023) and *msi*^*2*^ (31%, *N* = 100; Fisher’s exact test, *p* = 0.0002) mutant FSC clones at 21 days PCI was observed. Ovarioles were acquired and imaged randomly from a minimum of 18 heat shocked flies of each genotype. **C** Single-plane confocal micrographs showing Traffic Jam (Tj, red) positive, GFP-negative clones generated by Flp-FRT. A FSC clone (pink arrowhead) and FC clone (pale green arrowhead)) are depicted. Scale bars, 10 µm. Dotted line represents the Fas3 expression boundary.

Morphological analysis of *msi* mutants uncovered a function for Msi in region 2–3 somatic cells of the germarium, but we have not uncovered any evidence to support a requirement for the shorter isoforms of Msi in female germ cell maintenance or differentiation. Given the high level of Msi antibody labelling observed in the GSCs (Fig. 1B, C), we expected clonal analysis to reveal a functional requirement for Msi in the maintenance of GSCs comparable with previous observations in the testis [14]. Therefore, we generated loss of function GSC clones marked by the absence of GFP utilising two different *msi* mutant alleles, *msi*^*1*^ and *msi*^*2*^, and measured whether the frequency of mutant GSC clones could be maintained over time. At 7 days post clone induction (PCI), the percentage of germaria containing at least one control GSC clone (56.7%, *N* = 97) was not significantly different to the percentage containing at least one *msi*^*1*^ (54.16%, *N* = 96) or *msi*^*2*^ (55.67%, *N* = 97) mutant clone (Fig. 3A). At 21 days PCI, no significant reduction in the percentage of germaria containing control (54.16%, *N* = 97), *msi*^*1*^ (50.98%, *N* = 102) or *msi*^*2*^ (53.00%, *N* = 100) GSC clones was observed (Fig. 3A). These findings surprisingly revealed that, unlike the testis, there is no intrinsic requirement for the shorter Msi isoform/s in the maintenance of GSC identity in the *Drosophila* ovary.

Since we discovered a functional requirement for Msi in somatic cells of the germarium to regulate cyst morphogenesis, we sought to determine whether Msi may play a role in the regulation of FSCs, which also reside in this region. The percentage of germaria containing at least one Tj-positive but GFP-negative *msi*^*1*^, *msi*^*2*^ or control layer 1 FSC clone at the 2a/b boundary was compared at 7- and 21-days PCI (Fig. 3C). After 7 days PCI, at least one layer 1 control FSC clone was observed in 58.76% (*N* = 97) of germaria, not significantly different to the percentage of germaria containing at least one *msi*^*1*^ (51.04%, *N* = 96) or *msi*^*2*^ mutant clone (57.73%, *N* = 97) (Fig. 3B). By 21 days PCI, the percentage of germaria containing at least one control FSC clone remained relatively unchanged (57.29%, *N* = 96). In contrast, there was a significant reduction in germaria containing at least one layer 1 *msi*^*1*^ (29.41%, *N* = 102; Fisher’s exact test *p* = 0.0023) or *msi*^*2*^ (31.00%, *N* = 100; Fisher’s exact test *p* = 0.0002) mutant FSC clones compared to 7 days PCI (Fig. 3B), revealing a requirement for Msi in layer 1 FSC maintenance. These data demonstrate an isoform specific requirement for Msi in germarial somatic cells to support cyst morphogenesis and maintain FSC identity.

### msi mutant FSC clones do not express the cell death marker Dcp-1 but display aberrant morphology

**Fig. 4:**
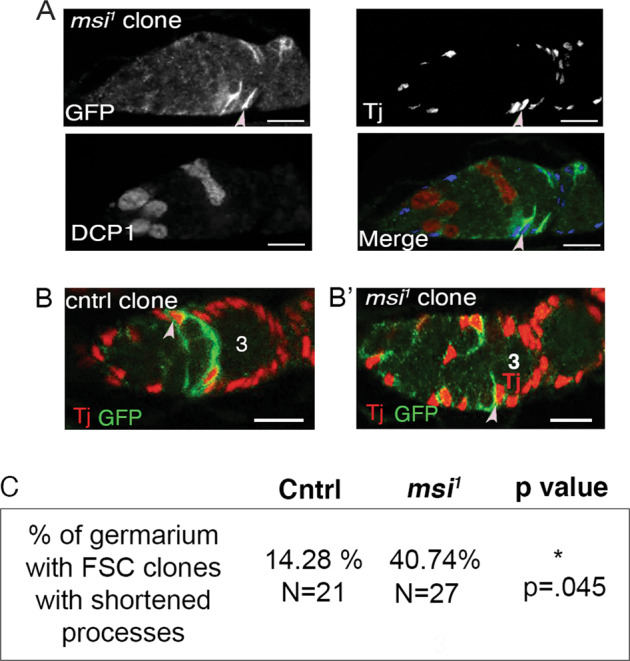
*msi* mutant FSC clones do not express Dcp-1 but display aberrant morphology. **A** Single-plane confocal micrographs showing Tj-positive (blue in merged panel) and GFP-positive MARCM *msi* null FSC clone (green in last panel). This image depicts Dcp-1 positive germ cells dying as a control for Dcp-1 antibody labelling. **B** Single-plane confocal micrograph showing a control, layer 1 GFP-positive FSC clone (light pink arrowhead) with an axon-like projection extending across the germarium. **B**’ Single-plane confocal micrograph showing a *msi* null, layer 1 GFP-positive FSC clone (light pink arrowhead) with a shortened, aberrant projection. **C** Table represents the % of control (*N* = 21) and *msi*^*1*^ (*N* = 27) mutant 10 day old FSC MARCM clones with shortened axon-like processes. *p* value (0.045) was calculated using Fisher’s exact test. Ovarioles were acquired from a minimum of 15 heat shocked females for each genotype. Scale bars, 10 µm. Three represents a region 3/stage 1 egg chamber.

Morphological analysis of *msi* mutants revealed a cyst collision phenotype in approximately 1/3 of ovaries examined (Fig. 2D, E). In region 2a/2b, somatic cells directly anterior and adjacent to layer 1 FSCs have been described as proliferatively active layer 2–3 FSCs [12]. Also in this region reside posterior ECs [7, 8]. In our clonal analysis, we observed negatively marked region 2a-2b control somatic cell clones corresponding to FSC layers 2 and 3 at both 7- and 21- days PCI (Supplementary Fig. 5A, B). While the percentage of germaria containing somatic control clones in this region remained similar at both 7- and 21- days PCI, a significant difference in the percentage of germaria containing at least one layer 2 or 3 *msi*^*1*^ (Fisher’s exact test *p* = 0.03) or *msi*^*2*^ clone (Fisher’s exact test *p* = 0.01) was observed (Supplementary Fig. 5B). Moreover, some *msi* mutant clones appeared to be aberrantly positioned on the outer edge of the ovary (Supplementary Fig. 5C). Loss of *msi* somatic cell clones led to the question of whether mutant clones were dying. To test this, we generated GFP-labelled *msi*^*1*^ mutant FSC clones by MARCM (Mosaic Analysis with a Repressible Cell Marker) to visualise whether GFP marked clones co-expressed the cell death marker Dcp-1 (*Drosophila* caspase-1). Of the 27 *msi*^*1*^ mutant layer 1–3 FSC clones analysed 12 days PCI, none expressed Dcp-1, suggesting that *msi* mutant clones were not being lost due to cell death (Fig. 4A). However the morphology of CD8::GFP labelled *msi*^*1*^ FSC clones differed from wild-type counterparts. Layer 1–3 FSCs have been shown to extend processes, or axon-like projections, across the entire germarium [12, 41]. Of the 27 germaria carrying a GFP marked *msi*^*1*^ mutant FSC clone, 11 displayed aberrant, shortened projections (Fig. 4B, C). In comparison, we only observed 3 control GFP clones with aberrant extensions. Shortened processes in region 2a somatic cells is a hallmark of EC morphology [12] and it is possible that loss of Msi function could be causing some FSCs to adopt an EC fate. This would be consistent with the known intrinsic requirement of Msi to maintain stem cell self-renewal capabilities in *Drosophila* GSCs [14]. Unfortunately, we have been unable to test this hypothesis owing to a lack of identification of FSC-specific drivers and markers.

### Loss of Msi function in somatic cells causes cell cycle defects and an increase in dying germline cysts in region 2a/b of germaria

**Fig. 5:**
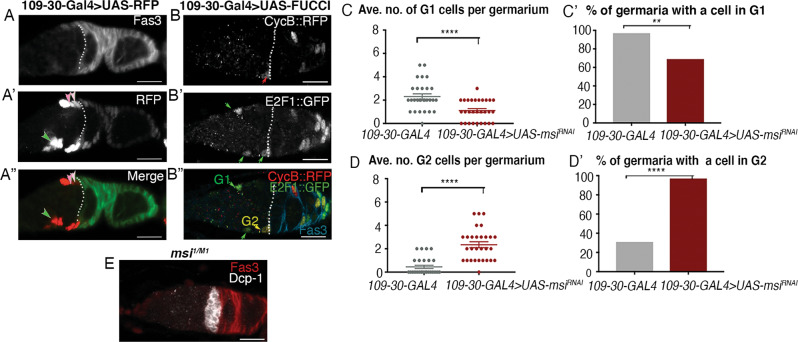
Loss of Msi function in somatic cells causes cell cycle defects and an increase in dying germline cysts in region 2a/b of germaria. **A**-**A**” Confocal micrograph (projection of 3 planes from a z stack) showing a representative image of a *109-30-Gal4* > *UAS-RFP* adult germarium. Dotted line represents the Fas3 expression boundary. A FSC in each of layers 1 and 2 (light pink and medium pink arrowheads respectively) and a posterior EC (green arrowhead) that are expressing RFP are labelled in (**A**’) and (**A)**”. **B** Confocal micrograph showing a representative image of a *109-30-Gal4* > *UAS*^*FUCCI*^ adult germarium. A CycB::RFP-positive cell (red arrow), E2F1::GFP positive cells (green arrows), A G2 cell (yellow arrow in **B**”) and G1 cells (green arrow in **B**”) anterior to the Fas3 expression boundary (dotted line) are labelled. **C** Scatterplot showing a significant difference in the average number of cells (±SEM) in G1 within region 2a–2b of germaria dissected from *109-30-Gal4* (2.31 ± 0.23, *N* = 29) and *109-30-Gal4* > *UAS-msi*^*RNAi*^ (1.18 ± 0.16, *N* = 29) (Welsh’s two*-*tailed *t* test *p* < 0.0001) adults. **C**’ Column graph showing a significant decrease in the % of *109-30-Gal4* > *UAS-msi*^*RNAi*^ germaria with at least one cell in G1 (69%; *N* = 29; Chi*-*square p = 0.005) when compared to *109-30-Gal4* germaria (97%). **D** Scatterplot showing a significant difference in the average number of cells (± SEM) in G2 within region 2a-2b of germaria dissected from *109-30-Gal4* (0.45 ± 0.14, *N* = 29) and *109-30-Gal4* > *UAS-msi*^*RNAi*^ (2.35 ± 0.25, *N* = 29) (Welsh’s two-tailed *t* test *p* < 0.0001) adults. **D***’* column graph showing significant increase in the % of *msi*^*RNAi*^*; 10930*^*Gal4*^ (97%; *N* = 29; Chi-square *p* < 0.0001) mutant germaria with the presence of at least one cell in G2 compared to control germaria (31%). Ovarioles were acquired and imaged randomly from a minimum of 8 adult flies. **E** Single-plane confocal micrograph of *msi*^*1/M1*^ ovary labelled with Dcp-1 (white) and Fas3 (red). Scale bars, 10 µm.

The aberrant morphology of *msi* mutant layer 1–3 FSCs is suggestive of differentiation defects, perhaps at the expense of proliferation. Because these FSCs have been defined as proliferatively active, we asked whether loss of Msi causes cell cycle defects. Therefore, we used the *Drosophila* Fluorescence Ubiquitin-based Cell Cycle Indicator (Fly-FUCCI) system to fluorescently label cells in G1, S and G2 phases of interphase [42] and determine whether *msi* somatic cells were cycling normally. In the fly FUCCI system, G1 is distinguished by GFP::E2F1 labelling in the absence of RFP::CycB. G2 cells, expressing both RFP and GFP, appear yellow, and cells in S phase are labelled by RFP alone. *109-30-GAL4* was used to drive a GAL4 responsive UAS-fly-FUCCI transgene (UAS-FUCCI) in somatic cells encompassing FSC layers 1–3 and escort cells abutting region 2a of the germaria [7] (Fig. 5A, B). Within the boundary of region 2a and region 2b (excluding mature follicle cells), Msi knockdown resulted in a significant reduction in the average number of somatic cells in G1 per germaria (Fig. 5C). Chi-square analysis also revealed a reduction in the proportion of *109-30-Gal4* > UAS-*msi*^*RNAi*^ mutant germaria containing at least one cell in G1 (*p* = 0.005; Fig. 5C’). Msi knockdown also resulted in a significant increase in the average number of cells in G2 per germaria compared to controls (*t* test *p* < 0.0001; Fig. 5D) and a significant increase in the total number of germaria exhibiting at least one somatic cell in G2 (Chi-square *p* < 0.0001; Fig. 5D’). Our results suggest that loss of Msi function from somatic cells within region 2a-2b of the germarium leads to a lag in G2 phase of the cell cycle and supports the overall finding that Msi is required to maintain the identity of somatic stem cells and to support the function of ECs in this region.

Our findings have established an isoform specific requirement for Msi in maintaining FSC fate and supporting germline cyst morphogenesis in early oogenesis. An accumulation of germline cysts in region 2–3 of the germarium, cyst collisions and the formation of compound egg chambers suggest that somatic cells fail to properly interact with, and support, the developing germline. Further evidence to support this comes in the way of analysis of cell death in *msi* mutants. We observed a twofold increase in the number of germline cysts that express the cell death marker Dcp-1 in *msi*^*1/M1*^ (11/31) mutant germaria compared to a *w*^*1118*^ controls (5/32) (Fig. 5E). Normally, cell death in region 2b of the germarium occurs sporadically in well fed flies (reviewed in [43]). In *msi*^*1/M1*^ mutants, 9 of the 11 dying cysts in *msi*^*1/M1*^ mutants were in region 2a/b, supporting the hypothesis that *msi* mutant somatic cells in this region are defective in providing the necessary signals to the germline to fully support germline cyst progression in early oogenesis.

### Losing Msi function from somatic cells results in mis-expression of Lamin C suggesting an altered differential potential of FSCs

**Fig. 6:**
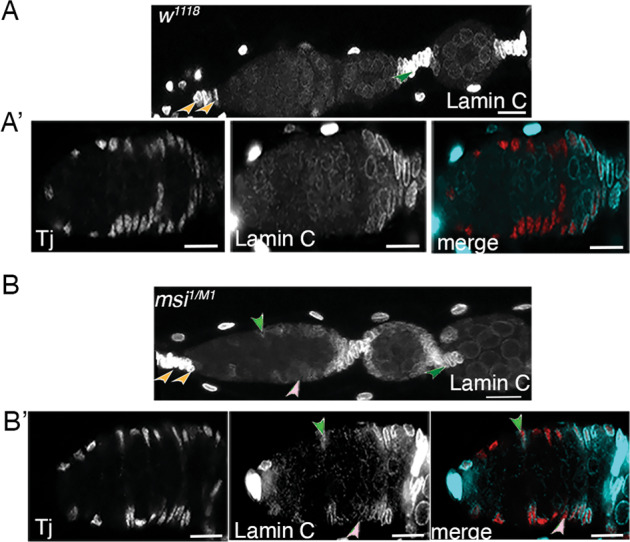
Losing Msi function from somatic cells results in mis-expression of Lamin C. **A**-**A**’ Representative confocal micrographs (projection of 3 planes from a z stack) showing normal Lamin C expression in TFs and CCs (orange arrowheads) and stalk cells (green arrowhead) in a wild-type germarium. **B**-**B**’ Confocal micrographs (projection of 3 planes from a z stack) showing up-regulation of Lamin C in somatic cells in germaria dissected from *msi*^*1/M1*^ adults. Ovarioles were dissected from a minimum of 5 adult flies and imaged randomly. A layer 1 FSC (light pink arrowhead) and posterior EC (green arrowhead) expressing Lamin C are labelled. Scale bars, 10 µm.

The separation of egg chambers relies upon correct differentiation of FSCs and their progeny. Egg chambers are separated by 5–8 flattened disc-like somatic stalk cells which differentiate from the pool of follicular precursor cells and connect the anterior end of a more mature egg chamber with the posterior end of a younger chamber [11]. Stalk cells, along with terminal filament cells and cap cells, express high levels of Lamin C (Fig. 6A). We asked whether stalk cell formation occurred normally in *msi*^*1/M1*^ mutants by labelling ovaries with anti-Lamin C. While Lamin C-positive stalk cells were observed in *msi*^*1/M1*^ mutant ovaries (Fig. 6B) and the number of stalk cells did not appear to be affected, we surprisingly observed an up-regulation of Lamin C expression in FSCs, FCs and some ECs in the germarium (Fig. 6B-B’). Up-regulation was consistently observed in region 2a/b (Fig. 6B), with occasional region 1 escort cells displaying increased Lamin C levels (Fig. 6B’). These results support the idea that loss of Msi function from somatic cells within this region results in altered differentiation potential of FSCs and that Msi is required to maintain somatic stem cell identity. Our results also point to a functional requirement for Msi in a specific subpopulation of ECs in region 2a/b to support germline cyst morphogenesis.

## Discussion

*Drosophila* Msi has known roles in determining fate outcome of ectodermal sensory organ precursors [36], photoreceptor [32] and crystal cell determination [44]. Msi is required for maintenance of spermatogonial GSCs [14] and modulates intestinal stem cell proliferation after radiation induced damage [18]. These studies highlight the importance of Msi in the context of cell fate determination and stem cell maintenance.

### Sex-specific differences in *Drosophila* RBP function for maintenance of GSC identity have been described

Our analysis has revealed that Msi isoforms exhibit divergent expression in the germline and soma of both the *Drosophila* testis and ovary. In each, the longer protein isoforms were only detected in somatic cells. Surprisingly we found no functional requirement for shorter Msi isoforms in GSC maintenance in the female despite being highly expressed in female GSCs and intrinsically required to maintain GSC identity in the testis [14]. Since Msi is an RBP and its target mRNAs remain unknown, this disparity could simply reflect differences in the genetic machinery required to regulate GSC identity in males and females. Sex specific differences in RBP function have certainly been described with other RBPs such as Pumilio, Nanos and Held Out Wings [45, 46].

### Msi regulation of *Drosophila* epithelial stem cells is consistent with the known function of its vertebrate orthologues

Despite not finding a requirement for short Msi isoforms in maintaining ovarian GSC function, we demonstrated that one (or more) of the longer isoforms is required to regulate the differentiation outcome of FSCs. *msi* null and hypomorphic mutant FSC clones were not maintained in the germarium since FSCs in layer 1 and the adjacent layers 2 and 3 were lost over time. Only the longer isoforms are expressed in somatic cells of the ovary, therefore demonstrating a divergence of Msi isoform function in a context-dependent manner for the regulation of different stem cell populations. The requirement for Msi to maintain the epithelial stem cell fate is consistent with the known function of its vertebrate orthologues. Msi-2 plays a critical role in hematopoietic cell fate and lineage bias [25]. Vertebrate Msi proteins have been shown to be associated with epithelial cell identity in several cancer types, most notably breast cancer [47] and are required for maintenance of quiescent intestinal stem cells [48]. The discovery that Msi is required for the regulation of an epithelial stem cell population in *Drosophila* provides a model to investigate mechanisms that underpin the maintenance of epithelial stem cells in a context-dependent manner.

### The requirement for Msi in a subpopulation of somatic cells in the *Drosophila* ovary likely reflects a requirement for Msi to bind target mRNAs in a context-dependent manner

An interesting aspect arising from our research was the discovery that Msi is required in a distinct population of somatic ECs to support early germline cyst progression in oogenesis. Several studies have revealed morphological and functional differences in ECs depending on their position within the germarium [3]. Recent scRNAseq analyses have demonstrated the existence of at least two EC subgroups [7, 9], with one study claiming as many as four subpopulations [8]. These analyses have uncovered functional differences between EC populations, with anterior ECs acting on GSCs and cystoblasts to support synchronous cell division, while more posterior ECs regulate soma-germline cell adhesion and the transition from 16-cell cyst-to-egg chamber formation. In our study we found no evidence for Msi function in anterior ECs. In *msi*^*1/1*^ mutant ovaries, early germline development progressed normally. Furthermore, the excess cysts that were observed in *msi* mutants was restricted to regions 2a/b and 3. Shi and colleagues (2021) observed a similar swollen germarial phenotype to that which we observed in mutant ovaries, including the accumulation of 8 cell cysts in germaria upon ablation of posterior ECs. In context of this recent literature, our results indicate a specific requirement for Msi function in a subpopulation of ECs and likely reflect the requirement for Msi to bind target mRNAs which have expression limited to somatic cells within this domain of the germarium.

### Loss of Msi from FSCs and pECs does not result in cell death, but cells exhibit cycling and differentiation defects

Our study has identified a functional requirement for Msi in FSCs and posterior ECs. The demarcation between ECs, FSCs and FSC progeny is not well characterised and remains controversial. scRNAseq has failed to distinguish a FSC population [7, 9]. One study has demonstrated that posterior ECs can convert to FSCs, at least under starvation conditions [10]. Others have demonstrated that FSCs can give rise to posterior ECs and even anterior ECs over time [7, 12]. Given the evidence that FSCs and posterior ECs are similar in their transcriptional profile and overlap in function, it is likely that the binding targets of Msi are the same in both cell types and Msi is required to maintain the fate and functionality of both cell types. Consequently, loss of Msi in these cells results in their inability to support germline cyst progression and fate interchangeability. Indeed, loss of Msi function from somatic cells in the 109-30-Gal4 expression domain, which encompasses both FSCs and posterior ECs, results in a lag in the G2 phase of the cell cycle and an up-regulation of a differentiation marker Lamin C. These cells are not lost from the germarium due to cell death, but mis-expression of Lamin C implicates a change in cell fate due to loss of Msi function.

### Similar to *Drosophila* Msi, differential roles for vertebrate Msi isoforms in development have been identified

Our study has identified Msi isoform specific requirements in stem cell maintenance in *Drosophila*. Mammalian Msi-2 is the closest Msi orthologue to *Drosophila* Msi and has four isoforms, all of which contain two RNA-recognition motifs (RRMs) but differ in the N-terminus or C-terminus [30]. This is comparable to *Drosophila* Msi, where the RRMs are conserved between the isoforms but proteins differ at the N-terminus (Supplementary Fig. 2). Recent studies have begun to highlight the different roles vertebrate Msi isoforms may play in tissue homoeostasis. One study has shown that a truncated Msi-2 isoform lacks regulatory phosphorylation sites and is overexpressed in multiple cancers [49]. Another recently has highlighted differential expression patterns of Msi-2 isoforms in triple-negative breast cancer (TNBC) and has demonstrated that downregulation of a predominant isoform (Msi-2a) is associated with TNBC progression [30]. Future studies into the role of *Drosophila* Msi isoforms in development will add insight into how specific isoforms can differentially regulate stem cell behaviour in a sex- and cell-specific manner.

## Materials and methods

### Fly strains

All flies were raised on standard molasses-based food at 25 °C except for Gal4 crosses, which were all carried out at 29 °C. Fly stocks used in this study obtained from the Bloomington stock centre (Indiana) include *w*^*1118*^ (BL5905)*, msi*^*1*^ (BL4160), *msi*^*2*^ (BL4161), Mi{MIC}msi^M101988^ (*msi*^*M1*^; BL33097), Mi{PT-GFSTF.2}msi^M100977-GFSTF.2^ (Msi-GFP; BL61750), 109-30-Gal4 (BL7023), UAS-mcD8::GFP (CD8::GFP) reporter on 1^st^ (BL5136) and 3^rd^ chromosomes (BL5130), UAS-FUCCI transgenes (BL55110, BL55111), *FRT82BUbi-GFP* (BL5188), Frt42D;FRT82B (BL8216) and *hh*-lacZ (BL5530). *frt82Bmsi*^*1*^ and *frt82Bmsi*^2^ were previously made in our laboratory [14]. MARCM82B flies (hsflp, UAS-GFP::CD8;; tubulin-GAL4, FRT82B tubP-GAL80) were a gift from the Quinn lab (Australia National University). The X chromosome *msi*^*RNAi*^ strain was obtained from the Vienna Drosophila Resource Centre (VDRC #11784). *tj*-GAL4 (DGRC104055) was obtained from Kyoto Stock Centre (Japan). c587-GAL4 and *Fax-GFP* lines were gifts from the Xie lab (Stowers Institute for Medical Research, Missouri, USA).

### Immunostaining and image analysis

Appropriately aged females were dissected in 1x PBS (diluted from a 20x PBS solution, Catalogue (Cat.) No. 97062-948, VWR Life Science), fixed for 20 min in 4% Formaldehyde (diluted from 16% ampule, Cat. No. 28908, ThermoFisher Scientific) in PBT (PBS + 0.2% Triton X-100 (Product No. 234729, Sigma)), washed for 3 × 10 min in PBT and then incubated for 45 min in PBTH (5% Normal Horse Serum, Cat. No. 26050070, ThermoFisher Scientific, diluted with PBT). Ovaries were then incubated overnight at 4 °C in primary antibodies diluted in PBT. Samples were washed a further 3 × 10 min in PBS before secondary antibody incubation was carried out for 2 h at room temperature in PBT. Samples were washed for a further 3 × 10 min before ovaries were mounted on slides in Prolong™ Gold Antifade Reagent with Dapi by Invitrogen (Cat. No. P36935, ThermoFisher Scientific). Antibodies used in this study include 1:10 rat anti-Msi (gift of H. Okano, Keio University), 1:20 mouse anti-Fas3 (7G10, Developmental Studies Hybridoma Bank (DSHB)), 1:40 mouse anti-Lamin C (LC28.26, DSHB), 1:100 rabbit anti-DCP1 (Cleaved *Drosophila* DCP-1 (Asp215) Cat. No. 9578 S, Cell Signalling technology), 1:100 goat anti-Vasa (dc-13, sc-26877, Santa Cruz Biotechnology), 1:2000 chicken anti-GFP (ab13970, Abcam), 1:500 rabbit anti-RFP (Cat. No. R10367, Invitrogen), 1:10 mouse anti-1B1 (1B1, DSHB), 1:1000 chicken anti-β galactosidase (ab134435, Abcam), 1:10,000 guinea pig anti-Traffic Jam (gift of Dorothea Godt, University of Toronto) and 1:500 mouse anti-C(3)G (gift of Scott Hawley, Stowers Institute of Medical Research, Kansas City). Secondary antibodies Donkey anti-Mouse Alexa Fluor 488 (Cat. No. A-21202), 594 (Cat. No. A-21203) and 647 (Cat. No. A-32787), Donkey anti-Rabbit Alexa Fluor 594 (Cat. No. A-21207), Donkey anti-Rat Alexa Fluor 488 (Cat. No. A-48269) and 594 (Cat. No. A-21209) were obtained from (ThermoFisher Scientific) and used at a dilution of 1:500. Donkey anti-guinea pig 594 (Cat. No 706-586-148), and donkey-anti chicken 488 (Cat. No. 703-545-155) were obtained from Jackson Immuno Research Labs and used at a dilution of 1:500. Images were acquired on Zeiss LSM800 or LSM880 confocal microscopes as serial optical sections (z-stacks) optimized to acquire overlapping sections. FIJI/ ImageJ was then used to process images and add scale bars. Adobe photoshop 2021 was used to compile figure panels.

### Mosaic analysis

Negatively marked GSC and FSC clones were induced by Flp-mediated recombination at FRT sites in 2–3 day old females of genotypes *hs*-Flp/+; FRT82B *msi*^*1*^/FRT82B *Ubi*-GFP, *hs*-Flp/+; FRT82B *msi*^*2*^/FRT82B *Ubi*-GFP, *hs*-Flp/+; FRT42D/ + ;FRT82B/FRT82B *Ubi*-GFP. Well-fed females were heat shocked in a water bath at 37 °C for 1 h, twice a day for 2 consecutive days. Daily heat shocks were conducted 8 h apart to aid recovery between heat shocks. Ovaries were dissected from 7 and 21 day old females (post heat shock) and stained with anti-GFP, anti-Traffic Jam and anti-Fas3. All GFP-negative but TJ positive clones at the 2a/2b boundary were counted as layer 1 FSC clones. All GFP-negative but TJ positive clones in the layers directly anterior and adjacent to layer 1 clones were counted as layer 2–3 clones. All GFP-negative but TJ positive clones anterior to layer 2–3 clones were counted as escort cell clones.

### MARCM analysis

GFP positive clones were generated in 2–3 old adult females of genotypes hsflp, UAS-GFP::CD8/+; tubulin-GAL4, FRT82B tubP-GAL80/FRT82B and hsflp, UAS-GFP::CD8 /+; tubulin-GAL4, FRT82B tubP-GAL80/FRT82B *msi*^*1*^. Females were subject to a 45 minute heat shock in a 37 °C waterbath, twice a day, for two consecutive days, with sufficient time in between heat shocks to ensure recovery. Ovaries were dissected at 10 days post heat shock and stained with anti-GFP, anti-Traffic Jam and anti-DCP1, and imaged as previously described. FIJI was used to analyse the images and the Fisher’s exact test in Prism 9 for Mac OS was used to calculate p values.

### FUCCI analysis

Full genotypes of flies used in FUCCI analysis include 109-30-Gal4/UASp-GFP::E2f, UASp-mRFP1.NLS::CycB; *+/+* (control) and *msi*^*RNAi*^/+; 109-30-Gal4/+; UASp-GFP::E2f, UASp-mRFP1.NLS::CycB. Flies were raised at 25 °C and shifted to 29 °C upon eclosion for 3 days. Well-fed females were dissected and stained for detection of GFP and RFP. Serial overlapping optical sections were analysed in FIJI, with DAPI used in conjunction with the ROI manager to make sure to not duplicate cell counts. The minimal brightness threshold utilised for the RFP and GFP channels was 100.

### Statistics

Statistical analyses were performed using Prism 9 for Mac OS. p-value calculations for all statistical analyses are noted in figure legends. All scatterplots are graphed showing the mean ± SEM. For Fig. 2, Welsh’s two-tailed *t* tests were used to calculate the p values between genotypes *msi*^*1/+*^ (*N* = 20 ovarioles) and both *msi*^*1/1*^ (*N* = 28 ovarioles) and *msi*^*1/2*^ (N = 22 ovarioles) and between genotypes C587-GAL4 > + (*N* = 18 ovarioles) and C587-GAL4 > msi^RNAi^ (*N* = 20 ovarioles). Ovarioles were dissected from a minimum of 8 adult females from each genotype and ovarioles were imaged randomly over two separate sessions. For Fig. 3, Fisher’s exact test was used to calculate the p values of the frequency of GSC and FSC clones present in ovarioles dissected at 7 days post heat shock from genotypes *frt82B* (control, *N* = 97 ovarioles), *frt82Bmsi*^*1*^ (*N* = 96 ovarioles) and *frt82Bmsi*^*2*^ (N = 97 ovarioles) and 21 days post heat shock from *frt82B* (control, *N* = 96 ovarioles), *frt82Bmsi*^*1*^ (*N* = 102 ovarioles) and *frt82Bmsi*^*2*^ (N = 100 ovarioles). Ovarioles were acquired from a minimum of 18 heat shocked flies of each genotype. For Fig. 4, Fisher’s exact test was used to calculate the p value comparing genotypes *frt82B* (control, *N* = 21 ovarioles) and *frt82Bmsi*^*1*^ (*N* = 27 ovarioles). Ovarioles were acquired from a minimum of 15 heat shocked females for each genotype. For Fig. 5, Welsh’s two-tailed *t* tests were used to calculate the p values comparing the average no. of cells in G1 and G2 in genotypes *109-30-Gal4* (*N* = 29 ovarioles) and *109-30-Gal4* > *UAS-msi*^*RNAi*^ (*N* = 29 ovarioles). Chi-square test was used to compare the % of germaria with cells in G1 or G2 from both genotypes. Ovarioles were acquired and imaged randomly from a minimum of 8 adult flies.

## Supplementary information

Supplemental Material

## Data Availability

All data generated or analysed during this study are included in this published article and in its supplementary information file.
